# Genome sequence of bacteriophage AmiCi24 isolated using *Arthrobacter globiformis*

**DOI:** 10.17912/micropub.biology.001824

**Published:** 2025-11-29

**Authors:** Paige Baldwin, Michael Bennett, Evangelia Buonamici, Hanan Isik, Niran Isik, Vienna Li, Mia Mazakas, Aaryan Modi, Nikita Muppoor, Kylie Oliver, Vidhi Patel, Kylie Tabak, Laura Weber, Jamie Yu, Matthew Farber, Marina Bogush

**Affiliations:** 1 Department of Biological and Biomedical Sciences, College of Science and Mathematics, Rowan University, Glassboro, NJ 08028, USA

## Abstract

AmiCi24 is a novel siphoviral bacteriophage that was isolated from a soil sample collected in Sewell, NJ, USA using *Arthrobacter globiformis* B-2979 as the host. AmiCi24 has a genome consisting of 38,466 base pairs that encodes 68 predicted protein-coding genes. Based on gene content, AmiCi24 is assigned to actinobacteriophage cluster AS and subcluster of AS3 and is predicted to be temperate.

**Figure 1. Characterization of AmiCi24 phage morphology:**
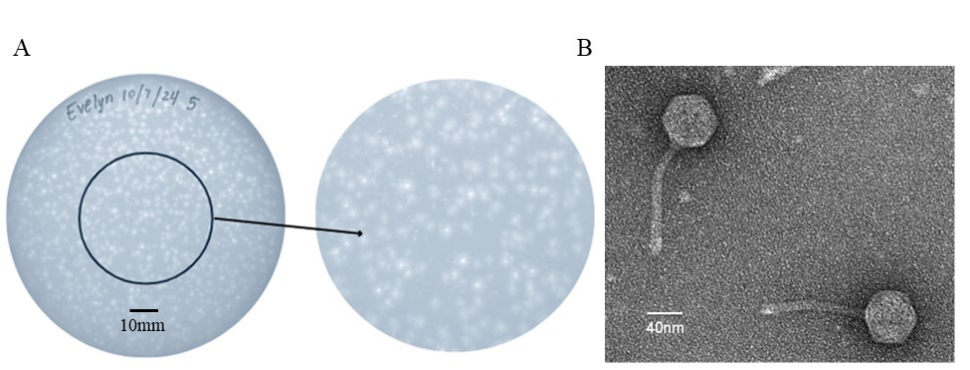
A) Plaques of AmiCi24 forming small bullseyes with clear centers and turbid halos. B) Negative stain (1% uranyl acetate) TEM micrograph of AmiCi24 obtained using a Hitachi HT7800 TEM, revealing a siphovirus morphology.

## Description

Bacteriophages, which are viruses that infect and replicate in bacteria, are finding increasing practical uses in medicine, industry, and food production. For example, phage therapy may provide an alternative to antibiotics in treating antibiotic-resistant bacterial infections (Abril et al., 2022; Hatfull et al., 2022; Mattila et al., 2015), and phages may be beneficial in food production as an alternative to pasteurization and chemical sterilization (Garvey, 2022). The study of bacteriophages may also elucidate mechanisms underlying natural selection of bacteria (Naureen et al., 2020).

To contribute to the discovery and characterization of novel phages, student researchers at Rowan University, through the Science Education Alliance-Phage Hunters Advancing Genomics and Evolutionary Science (SEA-PHAGES) program by the Howard Hughes Medical Institute (HHMI), report the discovery of a novel bacteriophage, AmiCi24. AmiCi24 was isolated from soil in Sewell, NJ (GPS coordinates: 39.75017 N, 75.11692 W). A damp and sandy sample of soil was collected using an entrenching tool. The soil sample was then incubated in peptone yeast calcium (PYCa) liquid medium and shaken at 250 rpm for one hour at 30°C. The suspension was spun and the supernatant was filtered through a 0.22-μm-pore-filter. The recovered filtrate was then inoculated with *Arthrobacter globiformis* B - 2979 and incubated with shaking at 30°C for 48 hours. The resulting culture was then filtered and an aliquot of the filtrate was plated in PYCa top agar with *Arthrobacter globiformis* B - 2979 (Zorawik et al., 2024). After 48 hours at 30°C, AmiCi24 formed small bullseye plaques with a diameter of approximately 3 mm based on measurements of 12 plaques. ( Fig. 1A ). After three rounds of phage AmiCi24 purification (picking plaques at least 1 cm away from other plaque), a high-titer lysate (1.25x10 ^9^ pfu/ml) was prepared and used for transmission electron microscopy (TEM) with negative staining (uranyl acetate 1%), revealing a siphovirus morphology with an icosahedral capsid 60 nm +/- 2 nm (n=3) in width and a long flexible tail 120 nm +/- 2 nm (n=3) in length measured in reference to the scale bar using a ruler ( Fig. 1B ).

Genomic DNA of AmiCi24 was extracted from a lysate with the Norgen Phage DNA Isolation Kit (Norgen Biotek Corporation). The extracted DNA (130 ng/μl) was then prepared for sequencing using the NEB Ultra II Library kit and sequenced on an Illumina NextSeq 1000 (XLEAP-P1 kit) to yield 1,989,938 100-base reads, providing an approximate shotgun coverage of 5014x across the genome. Raw reads were trimmed with cutadapt 4.7 (using the option: –nextseq-trim 30) and filtered with skewer 0.2.2 (using the options: -q 20 -Q 30 -n -l 50) prior to assembly (Martin 2011, Jiang et al., 2014, Wick et al., 2017, Gordon et al., 1998). The AmiCi24 genome is 38,466 base pairs long and features 3' single-stranded overhangs at its termini consisting of 12 bases in length with the specific sequence 5'-GAGTTGCCGGCA. The GC content of the genome is 66.1%, typical of Arthrobacter bacteriophages (Klyczek et al., 2018, Russell & Hatfull, 2017). Based on gene content similarity of at least 35% to phages in the Actinobacteriophage database, PhagesDB, AmiCi24 was assigned to cluster AS, subcluster AS3 (Russell & Hatfull, 2017; Pope et al., 2017).

The genome was annotated by Glimmer v3.02 (Delcher et al., 2007) and GeneMark v2.5p (Besemer & Borodovsky, 2005) using DNA Master v.5.23.6 ( https://phagesdb.org/DNAMaster/ ) and Phage Evidence Collection and Annotation Network ( https://discover.kbrinsgd.org/evidence/summary ). Starterator v1.2 (http://phages.wustl.edu/starterator/) was used to refine gene start sites. Phamerator (Cresawn et al., 2011), using Actino_draft database v578, BLAST, using the Actinobacteriophage and NCBI non-redundant databases (Altschul et al., 1990), and HHPRED, using the PDB_mmCIF70, Pfam- v.36, and NCBI Conserved Domains databases (Söding et al., 2005) were used as comparative tools to identify putative functions of protein-coding genes. Default settings and parameters were used for all databases and tools. AmiCi24 encodes 68 total putative protein-coding genes; 39 genes were assigned a putative function. No tRNA genes were identified using Aragorn v1.2.41 (Laslett & Canback, 2004) or tRNAscanSE v.2.0 (Lowe & Chan, 2016).

All but 13 genes located in the middle of the AmiCi24 genome are transcribed unidirectionally. Among the putative functions encoded by these 13 genes are HNH endonuclease, helix-turn-helix binding protein, tyrosine integrase, and immunity repressor. The presence of identifiable integrase and immunity repressor functions suggests AmiCi24 is able to establish lysogeny, which is supported by experimental data for another AS3 phage that encodes homologous integrase and immunity repressor (Jackson & Vega, 2025). Like other subcluster AS3 phages, the AmiCi24 genome contains genes involved in structure and assembly within the first third of the genome, while genes related to replication and recombination are scattered across the remaining two-thirds (Glaser & Monti, 2024). No tRNA genes were identified.

AmiCi24 is available at GenBank Accession No. PV915815 and Sequence Read Archive (SRA) No. SRX28150563
